# Effect of Water-Resistant Properties of Kraft Paper (KP) Using Sulfur Hexafluoride (SF_6_) Plasma Coating

**DOI:** 10.3390/polym14183796

**Published:** 2022-09-10

**Authors:** Pornchai Rachtanapun, Dheerawan Boonyawan, Rafael A. Auras, Gopinath Kasi

**Affiliations:** 1 Division of Packaging Technology, School of Agro-Industry, Faculty of Agro-Industry, Chiang Mai University, Chiang Mai 50100, Thailand; 2 The Cluster of Agro Bio-Circular-Green Industry (Agro BCG), Chiang Mai University, Chiang Mai 50100, Thailand; 3 Center of Excellence in Materials Science and Technology, Chiang Mai University, Chiang Mai 50200, Thailand; 4 Plasma and Beam Physics Research Facility, Department of Physics & Materials Science, Faculty of Science, Chiang Mai University, Chiang Mai 50200, Thailand; 5 School of Packaging, Michigan State University, East Lansing, MI 48824, USA

**Keywords:** contact angle, fluorine, surface roughness, water absorption, surface

## Abstract

Sulfur hexafluoride (SF_6_) plasma at different pressures, powers, and times was used to treat Kraft paper (KP) to enhance its water resistance. The KP was treated with SF_6_ plasma from 20–300 mTorr of pressure at powers from 25–75 Watts and treatment times from 1–30 min at 13.56 MHz. The prepared papers were characterized by contact angle measurement and water absorption. The selected optimum condition for the plasma-treated KP was 200 mTorr at 50 Watts for 5 min. Advancement with the change in treatment times (3, 5, and 7 min) on the physical and mechanical properties, water resistance, and morphology of KP with SF_6_ plasma at 200 mTorr and 50 Watts was evaluated. The changes in the chemical compositions of the plasma-treated papers were analyzed with an XPS analysis. The treatment times of 0, 3, 5, and 7 min revealed fluorine/carbon (F/C) atomic concentration percentages at 0.00/72.70, 40.48/40.97, 40.18/37.95, and 45.72/39.48, respectively. The XPS spectra showed three newly raised peaks at 289.7~289.8, 291.5~291.7, and 293.4~293.6 eV in the 3, 5, and 7 min plasma-treated KPs belonging to the CF, CF_2_, and CF_3_ moieties. The 5 min plasma-treated paper promoted a better interaction between the SF_6_ plasma and the paper yielded by the F atoms. As the treatment time for the treated KPs increased, the contact angle, water absorption time, and Cobb test values increased. However, the thickness and tensile strength did not show remarkable changes. The SEM images revealed that, as the treatment time increased, the surface roughness of the plasma-treated KPs also increased, leading to improved water resistance properties. Overall, the SF_6_ plasma treatment modified the surface at the nano-layer range, creating super-hydrophobicity surfaces.

## 1. Introduction

Kraft paper (KP) is a cellulose-fiber-based material. It is predominately used in the packaging industry for transpositions. Further, KP has better recyclability, renewability, reusability, and biodegradability properties, leading to an environmentally friendly material compared to synthetic polymer [1,2,3]. It is used in packaging because of its high elasticity and high-tear resistance property. KP is a highly durable material, making it suitable for more industrial and heavy-duty applications, such as shopping, heavy-duty bags, and corrugated boxes. A corrugated box is a three-layer structure consisting of the following: (i) an outer liner (flat skin of outer side), (ii) a fluting medium (shaped into a continuous wave on the inner side), and (iii) an inner liner (flat skin of inner side). KP virgin fiber material is used for the outer side, which provides better paper strength and is also easier to print on. Corrugated fiberboard is a material consisting of a fluted corrugated sheet and one or two flat liner boards. It contributes to material strength for stacking and resistance to crushing. However, modification of flute size is helpful for enhancing protection. Larger flutes promote strength and cushioning. Smaller flutes better enhance the printability and foldability. Test paper is a recycled KP used for the inside liner in the corrugated boxes due to their smaller strength than the outside of the KP [4]. Notably, corrugated board is used in the packaging field in the form of corrugated boxes and paper buckets [5,6]. Corrugated board has the best recycling performance of any packaging material in the UK, with an overall rate of over 80% recycling [2]. Corrugated board boxes make up about 80% of the total amount of paper packaging in the US. [5] In 2019, the Thailand corrugated packaging market was valued at USD 1405.73 million, and it is anticipated to grow to USD 2283.4 million by 2025 [7]. Due to their better structure and strength properties, most corrugated boxes act as the backbone of e-commerce [8,9]. The benefits include the impacts strength, renewability, low cost of production, light weight, and the fact that they are easy to modify to suit product packaging needs [10]. However, corrugated boxes have limited water resistance because corrugated materials are hydrophilic, and moisture content changes based on the moisture content of the air. Higher humidity causes the paper to have a higher moisture content and lowers resistance [11]. Standard technologies used for this purpose includes plastic coatings, wax coatings, and additives such as polyamine resin, polyethylene, polyvinyl chloride, and ethylene-vinyl alcohol copolymer [6,12]. However, these coatings are difficult to reuse and eliminate during recycling. Moreover, separating them from the corrugated outer layers is an expensive process, and these are causes of environmental issues [12].

Plasma is a quasi-neutral ionized gas, and it is primarily composed of ions, photons, and free electrons. These charged and neutral particles exhibit collective behavior [13,14,15]. It is induced by applying the appropriate power supply and frequency of energy to suitable gas, flow, and pressure [16]. The surface modification of material by a plasma process or by plasma applications, such as reactions, changes the morphology of nanoparticles and demonstrates the advantages of plasma processes. Plasma is environmentally friendly. After plasma treatment, surface roughness changes, increasing water-resistant properties [17,18]. Generally, argon (Ar) gas is used for plasma treatment. However, sulfur hexafluoride (SF_6_) gas produces fluorination on polymer surfaces, leading to a better hydrophobic surface. Rachtanapun et al. [13] reported the hydrophobicity of treated methylcellulose film, increasing the water-resistant properties using a SF_6_ plasma treatment. Paosawatyanyong et al. [19] reported that the lotus effect was observed in the SF_6_ plasma-assisted surface fluorination of polyethylene terephthalate (PET) fabrics. Plasma treatment increased the contact angle of the PET surfaces through increases in pressure, radio frequency power, and treatment time. The same research group investigated changes in the gas types of SF_6_, O_2_, N_2_, and Ar plasma treatments and revealed that the contact angle measurements increased for the SF_6_ plasma-treated PET samples compared to the other gases [16]. Recently, researchers have focused on plasma coatings to improve the surface modification of polymers [13,16,19,20,21]. Based on these works, a SF_6_ plasma coating was reported to greatly improve the hydrophobicity success of nano-coating on polymer surfaces due to the impact of the fluorine/carbon (F/C) ratio [21].

This work deals with Kraft paper type CA125 treated with SF_6_ plasma at varying gas pressures, powers, and durations of plasma treatment time. The screening of physical properties (grammage, thickness), mechanical properties (tensile resistance, folding endurance), water resistance, surface morphology, and the compositional ratio of F/C after plasma treatment is investigated.

## 2. Materials and Methods

### 2.1. Preparation of Kraft Paper for Plasma Treatment

First, 6 cm × 15 cm test Kraft paper (KP) type CA125 samples were cut. Then, the papers were conditioned and kept in desiccators containing a temperature of 27 ± 1 °C and a relative humidity (RH) of 65 ± 2 percent according to ISO standard 187:1990 (E) for 24 h. The papers used for plasma treatments with low temperatures were kept in chambers with a radio frequency power of 13.56 MHz. The pump pressure in these chambers was 1.7 ± 2 × 10^−2^ mTorr, and the papers then went through argon (Ar) gas into the chambers. The papers were cleaned using a pressure of 100 mTorr power with 50 Watts for 5 min before plasma treatment with sulfur hexafluoride (SF_6_). The optimum conditions for improving the water resistance of KP by plasma treatment with SF_6_ included the following three variables: pressure (20, 50, 100, 150, 200, 250, and 300 mTorr), power (25, 50, and 75 Watts) and treatment time (1, 3, 5, 7, 10, 15, 20, and 30 min).

### 2.2. Water Contact Angle and Water Absorption Time

The samples were cut to 3 cm × 12 cm and allowed to sit at room temperature for 30 min. A droplet (40 μL) of distilled water was dropped on the surface of the paper using a microsyringe, and the value of the contact angle was recorded at 30 s with a photograph and calculated with AutoCAD version 2003. The absorption time was the length of time it took for a water droplet to pass through a treated sample. This experiment was conducted by modifying two variables: the effect of pressure and plasma treatment times.

### 2.3. Tensile Strength, Folding Endurance, and Cobb Test Analysis

The tensile strength of the untreated and treated papers was investigated according to ASTM D 828-97. The 15 × 150 mm (W × L) 10 pieces of the test papers were cut along the cross-direction (CD) and machine-direction (MD) lines. Using the test paper holder, the test pieces were inserted into an A H1KS Universal Testing Machine (Tinius Olsen, Horsham, PA, USA), and the specimen length of the distance between the specimen gripping zones was measured at a distance of 100 mm. The time was set for the specimen and grip separation speeds at 25.4 mm/min. The average tensile strength was recorded. The folding endurance was tested using the TAPPI T220 sp-16 method. It was tested using a GOTECH model GT-6014-A instrument (Gotech Testing Machines Inc., Taichung, Taiwan). Briefly, 10 15 × 150 mm pieces of the test papers were cut along the CD and MD lines. Subsequently, the papers were inserted into the specimen holder on the tester, the distance of the pointer was adjusted to 70 mm, and the weight was set at 1000 g. The papers were tested until they broke. The average folding tolerance was reported by folding and then folding back, counting as one turn. The folding strength was quoted as the number of double folds. The Cobb test followed, using the TAPPI T441 om-90 method. Shortly, 125 × 125 mm test papers were cut, and the sample weights were measured before testing. A piece of paper was placed on an absorption test kit. After adding 100 mL of distilled water to the test kit’s ring, a timer was started. The test was performed for 120 s. After the test, the test paper was weighed once more to determine its final weight. The average percentage of water absorption was recorded after a total of 5 repeat tests.

### 2.4. Morphology and Structural Analysis

A scanning electron microscope (SEM) with an acceleration voltage of 3 kV was used to analyze the morphologies of the untreated and treated papers. The surface element content analysis was carried out with an X-ray photoelectron spectroscopy instrument (XPS, AXIS Ultra XPS spectrometer, KRATOS analytical, Manchester, UK). The parameters for XPS were set as a monochromatic Al K X-ray at 150 W anode power, survey spectra from 0 to 1200 eV with a pass energy of 80 eV for a survey, and 20 eV for core-level spectra. The chemical alterations in the paper samples following the plasma treatments were evaluated using two specimens of each group, and each specimen was analyzed for two sampling points. Characteristic oxygen (1s), carbon (1s), and fluorine (1s) peaks were searched, and the spectra were charge-corrected for the C–C/C–H peak (binding energy 284.7 eV). Spectra were evaluated with Casa XPS code software (Casa Software Ltd., Cheshire, UK).

### 2.5. Statistical Analysis

The data were analyzed using one-way analysis of variance (ANOVA), followed by Duncan’s multiple range test, and were determined with a significance level of *p* *≤* 0.05. SPSS software version 16.0 was used for the analysis. The different letter, e.g., ‘a’, ‘b’, ‘c’ or ‘d’ are statistically different (*p* < 0.05).

## 3. Results and Discussion

### 3.1. Selection of Plasma Gas

After cleaning, the surface of the paper with argon gas showed contact angle and water absorption time values of 96.0 ± 5.3° and 8.0 ± 1.5 min, respectively. The effect of Ar, SF_6_, and the combination of both gases influenced the treated KP for plasma at 100 mTorr, 50 Watts, and 5 min when evaluated by the contact angle and water absorption time, as summarized in Table 1. There was not a large difference when using pure SF_6_ or a combination of the Ar and SF_6_ gases, and the contact angle values were 117 ± 2.0° and 115 ± 3.5°, respectively. The water absorption time was shown to be over 200 min. As compared to the pure Ar and SF_6_ gases, the combination of both gases provided a better performance and was selected as the treatment gas.

### 3.2. Effects of Pressure, Power, and Treatment of Times Impact the Water Resistance Properties

**Figure 1:**
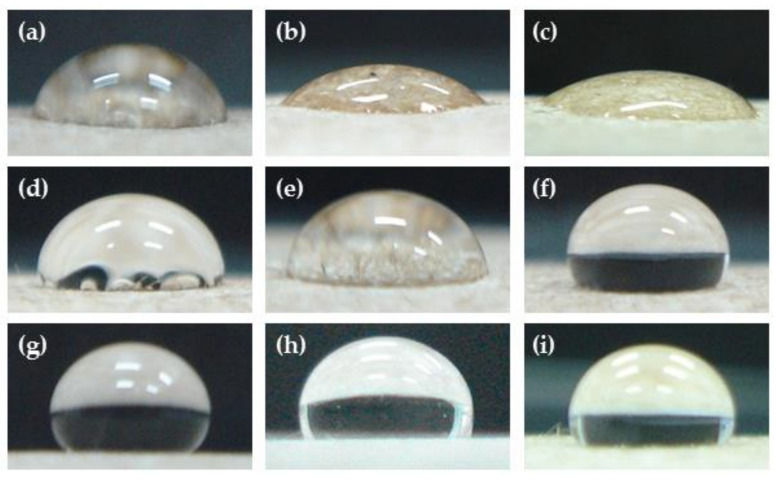
Effect of pressure on contact angles of water droplets placed (40 μL) at 200 mTorr and 50 Watts for (**a**) untreated, (**b**) Ar-treated, (**c**) 20 mTorr, (**d**) 50 mTorr, (**e**) 100 mTorr, (**f**) 150 mTorr, (**g**) 200 mTorr, (**h**) 250 mTorr, and (**i**) 300 mTorr.

**Figure 2:**
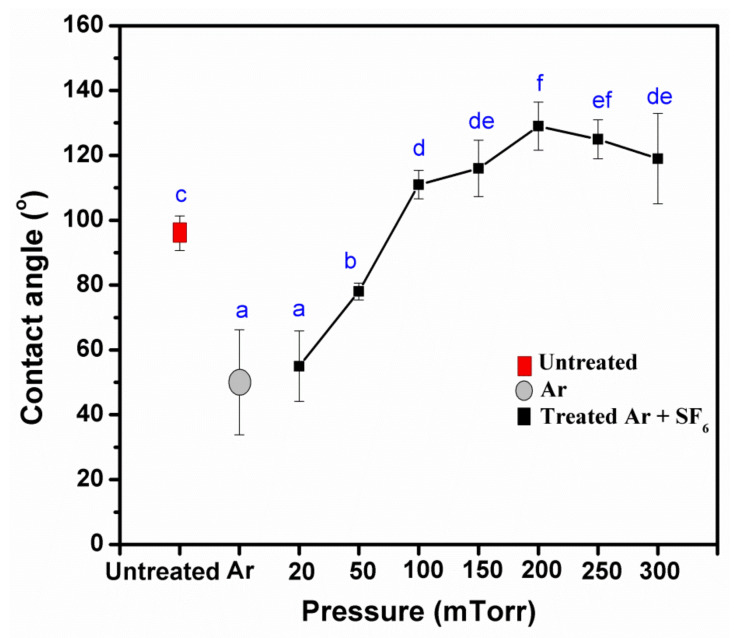
Effect of pressure on the contact angles of untreated and treated KPs with plasma at 50 Watts for 5 min. Note: values indicated with the same letters are not significantly different at *p* < 0.05.

**Figure 3:**
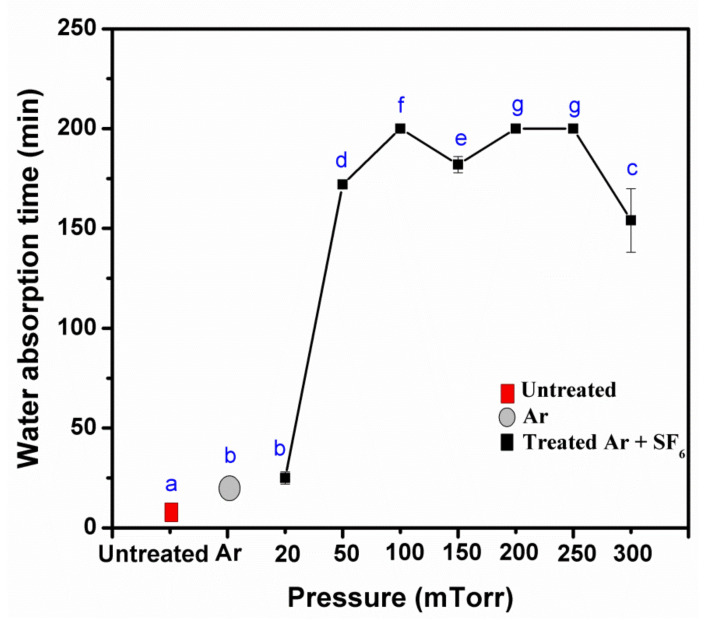
Effect of pressure on water absorption of untreated and treated KPs with plasma at 50 Watts for 5 min. Note: values indicated with the same letters are not significantly different at *p* < 0.05.

After plasma treatment, the KP contact angle increased due to an increase in the gas pressure, as shown in Figure 1 and Figure 2. In contrast, the contact angle decreased when the gas pressure was between 200 and 300 mTorr, maybe because the high-pressure condition of the ionization to plasma gas was not able to produce a uniform distribution [22]. Parallelly, the water absorption time also increased with the increased pressure. As a result, the highest pressures were observed at 200 and 250 mTorr (Figure 3). It was found that the contact angle and water absorption time of the paper were not significantly different *(p* < 0.05). Therefore, the optimum gas pressure was considered to be 200 mTorr.

**Figure 4:**
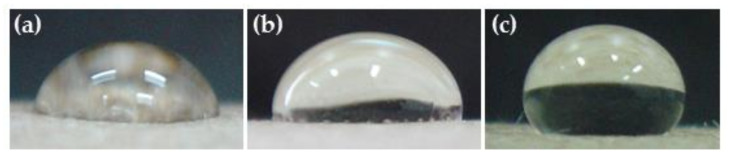
Effect of radio frequency (Watts) on water droplets placed (40 μL) at 200 mTorr for 5 min with (**a**) untreated, (**b**) 25 Watts, and (**c**) 50 Watts.

Power plays a key role in motivating plasma ionization. As the power increased, the contact angle and the water absorption time also increased (Table 2). These results match with previous findings [23]. However, the contact angle of a power application of 75 Watts could not be measured due to the reflection of power from the KP and the plasma burning-odor behavior. A radio frequency of 50 Watts was the highest power that improved the contact angle and the water absorption, from 111.0 ± 10.8° to 127.0 ± 6.1° (Figure 4) and from 177.0 ± 14.3 to >200.0 ± 0.0 min for KP. Hence, 50 Watts was determined as the optimum power treatment condition.

**Figure 5:**
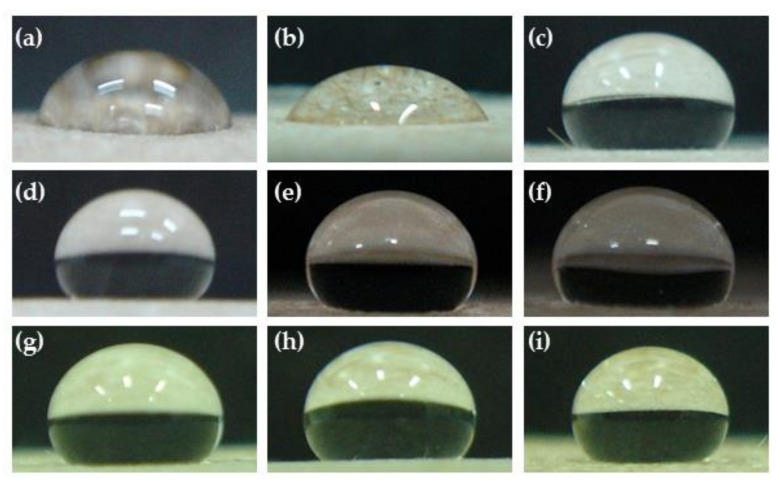
Effect of treatment time (minutes) on water droplets placed (40 μL) at 200 mTorr and 50 Watts for (**a**) untreated, (**b**) 1 min, (**c**) 3 min, (**d**) 5 min, (**e**) 7 min, (**f**) 10 min, (**g**) 15 min, (**h**) 20 min, and (**i**) 30 min.

**Figure 6:**
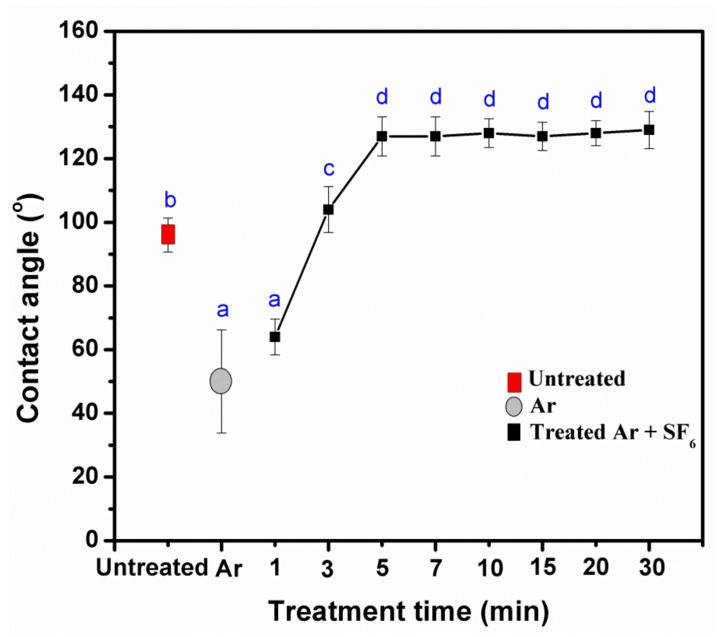
Effect of treatment time on the contact angles of untreated and treated KPs with plasma at 200 mTorr and 50 Watts. Note: values indicated with the same letters are not significantly different at *p* < 0.05.

**Figure 7:**
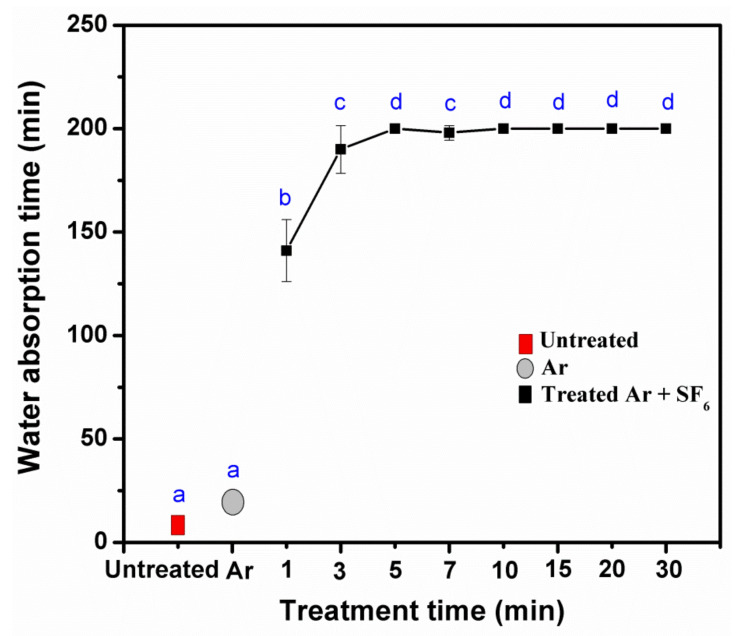
Effect of treatment time on water absorption of untreated and treated KPs with plasma at 200 mTorr and 50 Watts. Note: values indicated with the same letters are not significantly different at *p* < 0.05.

At different treatment times of plasma, 1min treated KP showed a decreased contact angle compared to the untreated KP. As per the increase in the time intervals of the plasma treatments, the water resistance of the KP consisted of the tendency of short times for increasing the contact angles of water droplets. Compared with untreated paper, the time was less than 3 min, and the contact angle increased up to 5 min. Afterward, the contact angle did not show a significant difference (*p* < 0.05) (Figure 5 and Figure 6), whereas the water absorption time of the paper increased as treatment time increased, and it was stable after 5 min of plasma treatment (Figure 7). Because of increased treatment times, the plasma highly reacted with the paper surface, beginning with a fixed-fiber paper at the point of reaction between the fluorine radicals. In contrast, increased time did not cause an increase in reactions, agreeing with previous reports [24,25]. Based on the observed results, mechanical, physical, morphological, water resistance, and structural analyses of the substrate were investigated with a fixed condition of pressure at 200 mTorr and power at 50 Watts to compare the effects of 3, 5, and 7 min treatment times. This study showed the treatment times of 3, 5, and 7 min using a radio frequency power of 50 Watts with a pressure of 200 mTorr.

### 3.3. Physical Properties of Treated KP

The grammage of the treated paper significantly decreased when compared with untreated paper. This weight loss could be attributed to the Ar treatment cleaning and heating [26]. Subsequently, the moisture content after treatment for 5 min showed a significant increase (*p* < 0.05) compared to untreated paper. Meanwhile, no differences were observed in the thicknesses of the treated and untreated KPs (Table 3) since the plasma treatment only modified the paper’s surface at the nanometer surface level [27].

### 3.4. Mechanical Properties of Treated KP

**Figure 8:**
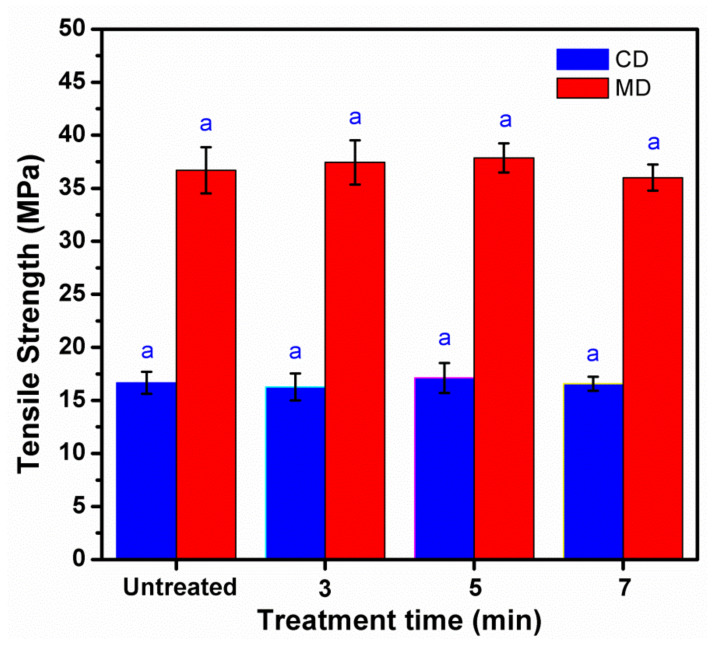
Effect of treatment time on tensile strength of untreated and treated KPs with plasma at 200 mTorr and 50 Watts (MD—machine direction, CD—cross direction). Note: values indicated with the same letters are not significantly different at *p* < 0.05.

**Figure 9:**
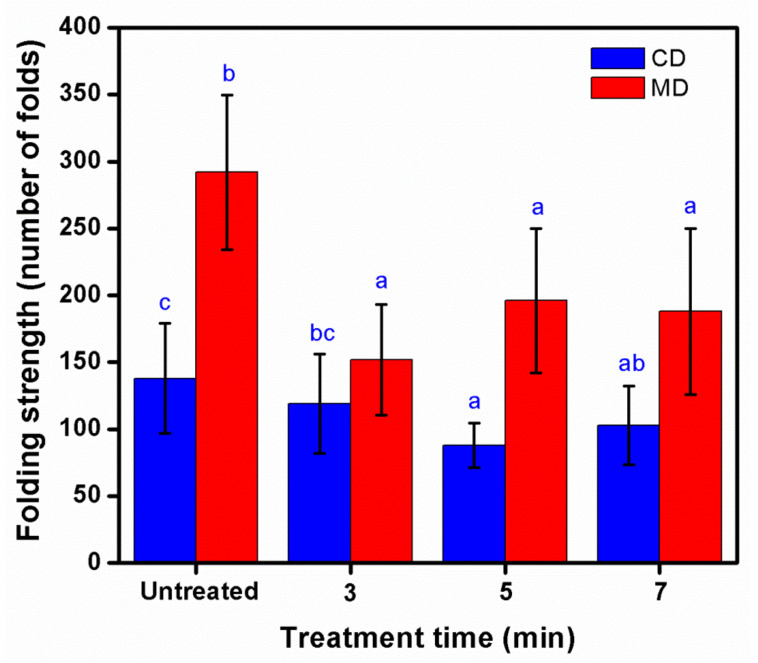
Effect of treatment time on folding strength (double folds) of untreated and treated KPs with plasma at 200 mTorr and 50 Watts (MD—machine direction, CD—cross direction). Note: values indicated with the same letters are not significantly different at *p* < 0.05.

After different treatment times of the KP, the tensile resistance did not change significantly compared to untreated paper (Figure 8). Appreciably, the MD of a paper had more tensile resistance than paper in the CD, as the paper fibers were extensively oriented in the MD direction. Jiankarn et al. [21] also reported that tensile properties did not show remarkable changes in KP during plasma treatment time increase. The folding strength decreased as time increased because the heat from the process led the paper to be more brittle (Figure 9). This trend was also observed in a previous study [21].

### 3.5. Water Resistance Properties of Treated KP

The Cobb test, as shown in Table 4, was used for testing the water resistance of the treated and untreated KPs. The untreated paper showed a high water sorption value at 124.0 ± 5.7 g/m^2^. As the treatment times (3, 5, and 7) increased for the KP, the water sorption values decreased to 34 ± 1.30, 28 ± 0.52, and 29 ± 4.35 g/m^2^, respectively. As expected, the modification of the nano-surface layer decreased the water sorption over three folding times for all the treatments. During the SF_6_ plasma ionization, the formation of fluorine atoms can replace the O and H atoms in the cellulose structure, causing the CF–CF groups to be distributed on a paper’s surface [21]. This modification of the surface layer at the nano-level covers the KP surface and reduces the penetration of water molecules into the pores. Therefore, the bonding energy decreases between the water and the paper, leading to an increase in the overall hydrophobic characteristics [23].

### 3.6. Morphology of Treated KP

**Figure 10:**
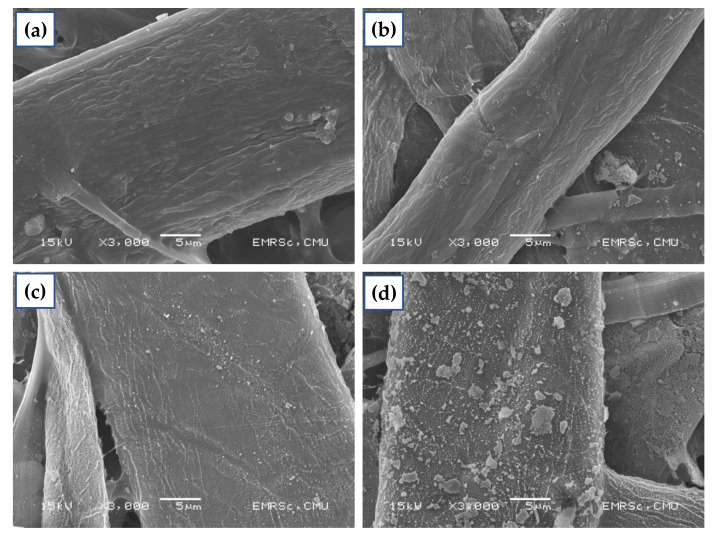
SEM images of (**a**) untreated, (**b**) 3 min, (**c**) 5 min, and (**d**) 7 min at 200 mTorr 50 Watts (3000×).

An SEM analysis provided information about the surface feature morphology changes [28,29]. As shown in Figure 10, the SEM images revealed surface morphologies for untreated and plasma-treated KPs. The untreated KP showed a smooth surface. After plasma treatment time, the surface roughness increased when compared to the untreated KP. Comparatively, 5 min of treatment exhibited a higher roughness because small, uniform modification of the surface improved the water resistance of the treated KP. This has been reported as a Lotus effect, and similar trends have been observed in previous reports [19,30]. This result correlated well with the reported contact angle and water sorption.

### 3.7. Chemical Composition of the Treated KP

**Figure 11:**
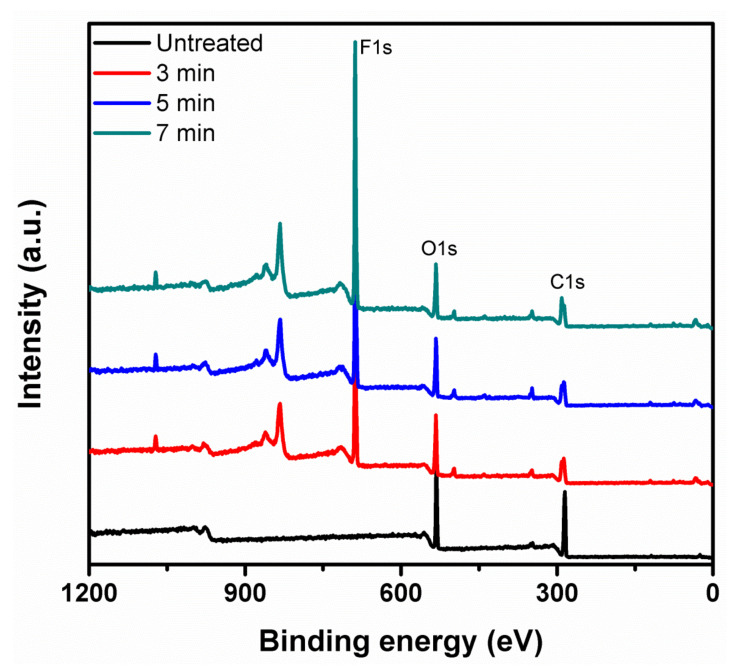
XPS analysis of full-survey spectra of untreated, 3 min, 5 min, and 7 min at 200 mTorr 50 Watts.

**Figure 12:**
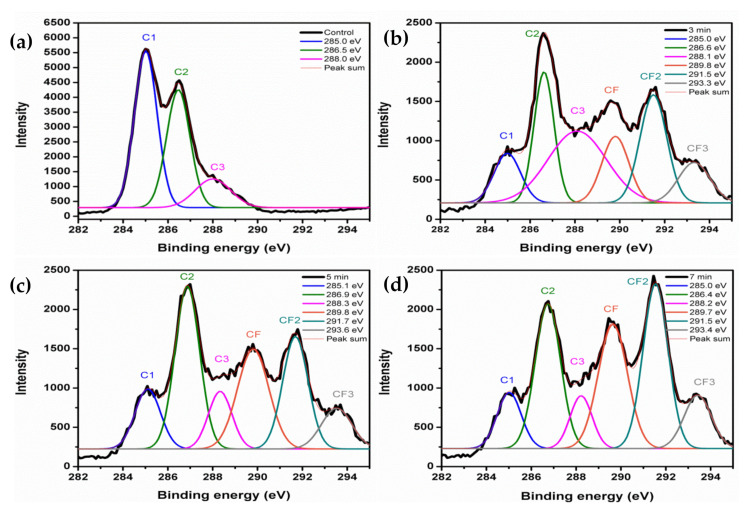
XPS analyses of C1s spectra of (**a**) untreated, (**b**) 3 min, (**c**) 5 min, and (**d**) 7 min at 200 mTorr 50 Watts.

The chemical compositions of the untreated and treated KPs were further explored with an XPS analysis. As shown in Figure 11, the XPS full-survey spectra of the untreated and treated samples showed *O1s* and *C1s* peaks. As expected, the spectra of the plasma-treated samples exhibited the *F1s* peak, confirming fluorine’s presence, whereas untreated and treated samples did not show a sulfur peak observed. This result matched well with a previous report [23]. The *C1s* XPS spectrum of untreated paper showed a peak at 285.0 eV corresponding to bonds from carbon bonded to other carbons (C–C). Subsequently, the peaks at 286.5 and 288.0 eV were attributed to bonds of C–O and C=O, respectively (Figure 12a), perfectly matched with the chemical composition of cellulose [31,32]. Treated KP XPS spectra revealed three newly raised peaks at 289.7~289.8, 291.5~291.7, and 293.4~293.6 eV detected in the 3, 5, and 7 min treatments and belonging to the CF, CF_2_, and CF_3_ moieties, respectively [22], as shown in Figure 12b–d. However, The *C1* spectrum of untreated KP showed high intensity, and it was suppressed by treatment times. At the same time, the *C2* spectrum peak predominantly emphasized at 3 and 5 min of treatment somewhat decreased at 7 min of treatment. After that, the treated KP showed newly appeared CF, CF_2_, and CF_3_, and these intensity peaks increased with time compared to untreated KP. The plots reveal that the trend in the C-to-F ratio decreased, and the F amount increased on the surface of the paper. This was caused because the hydrogen atoms were dissociated from the carbon atoms in C1s (C–C/C–H), and the oxygen (C2) and fluorine atoms (CF, CF_2_, and CF_3_) could be substituted for the hydrogen atoms in the cellulose structure during the plasma treatment. The treatment times of 0, 3, 5 and 7 min revealed fluorine/carbon (F/C) atomic concentration percentages at 0.00:72.70, 40.48:40.97, 40.18:37.95, and 45.72:39.48, respectively, confirming that the plasma treatment successfully bonded F onto the paper surface since, as treatment time increased, the F/C ratio increased. At 3 and 5 min, the F yield showed negligible differences. Hence, 5 min of treatment was suggested as an optimum condition for untreated paper. 

## 4. Conclusions

The water resistance of KP type CA125 was investigated by varying gas pressures, powers, and durations of plasma treatment. Variable treatments helped to determine the optimal condition for the water resistance ability: (i) a maximum pressure of 200 mTorr, (ii) a highest power of 50 Watts, and (iii) a treatment time of 5 min. In addition, the treatment was fine-tuned by changing the treatment times among 0, 3, 5, and 7 min. The grammage of treated papers decreased, while the thicknesses were no different than that of untreated paper. When the treatment time increased, folding endurance decreased while tensile strength remained unchanged. The machine direction of the treated KP had higher tensile strength than the cross direction. The moisture content after treatment decreased. After plasma treatment, the surface roughness at the nanometer level was observed, and the F/C atomic concentration percentage increased with an increase in the treatment time. Overall, SF_6_ gas that produced fluorination was successfully coated onto KP during 5 min of plasma treatment. In the treatment, F atoms replaced the O and H atoms in the cellulose structure, and this led to better water resistance behavior. SF_6_ plasma treatment can be used as an alternative approach to polymeric wax coating and can lead to minimizing environmental toxicity. Furthermore, this advancement in plasma treatment technology can be helpful to improve the hydrophobicity of targeted polymeric surfaces. Hence, this work helps to provide insights on improving the hydrophobicity of KP in various applications in the medical, food technology, and packaging fields.

## Figures and Tables

**Table 1:** Effect of argon and sulfur hexafluoride gas on the contact angles of untreated and treated KP with plasma at 100 mTorr, 50 Watts, and 5 min.

Gas	Radio Frequency (Watts)	Pressure (mTorr)	Contact Angle (°) *	Water Absorption Time (min) *
untreated	0	0	96 ± 5.3 ^a^	8 ± 1.5 ^a^
Ar	50	100	50 ± 16.2 ^b^	19 ± 0.6 ^b^
SF_6_	50	100	117 ± 2.0 ^c^	>200 ± 0.0 ^c^
SF_6_ + Ar	50	100	115 ± 3.5 ^c^	>200 ± 0.0 ^c^

* values in the same column with the same letters are not significantly different at *p* < 0.05.

**Table 2:** Effect of radio frequency (Watts) on the contact angles of untreated and treated KPs with plasma at 200 mTorr for 5 min.

Radio Frequency (Watts)	Contact Angle (°) *	Water Absorption Time (min) *
25	111.0 ± 10.8 ^a^	177.0 ± 14.3 ^a^
50	127.0 ± 6.1 ^b^	>200.0 ± 0.0 ^b^
75	n/d	n/d

* a, b—statistically different (*p* < 0.05), n/d: not detected.

**Table 3:** Effect of treatment time on grammage, thickness, and moisture content at 200 mTorr and 50 Watts.

Treatment Time (min)	Grammage (g/m^2^) *	Thickness (mm) *	Moisture (%) *
untreated	126.6 ± 0.2 ^b^	0.200 ± 0.010 ^a^	7.77 ± 0.14 ^b^
3	124.1 ± 0.6 ^a^	0.200 ± 0.010 ^a^	7.20 ± 0.15 ^ab^
5	124.0 ± 0.2 ^a^	0.200 ± 0.010 ^a^	7.06 ± 0.11 ^a^
7	123.8 ± 0.8 ^a^	0.200 ± 0.010 ^a^	7.24 ± 0.17 ^ab^

* values in the same column with the same letters are not significantly different at *p* < 0.05.

**Table 4:** Effect of treatment time on Cobb test of untreated and treated KPs with plasma at 200 mTorr and 50 Watts.

Treatment Time (min)	Cobb Test (g/m^2^) *
untreated	124 ± 5.7 ^c^
3	34 ± 1.30 ^a^
5	28 ± 0.52 ^b^
7	29 ± 4.35 ^b^

* values in the same column with the same letters are not significantly different at *p* < 0.05.

## Data Availability

The data presented in this study are available on request from the corresponding author.
